# The melanocortin receptors and their accessory proteins

**DOI:** 10.3389/fendo.2013.00009

**Published:** 2013-02-08

**Authors:** Shwetha Ramachandrappa, Rebecca J. Gorrigan, Adrian J. L. Clark, Li F. Chan

**Affiliations:** Centre for Endocrinology, William Harvey Research Institute, Queen Mary University of London, Barts and The London School of Medicine and DentistryLondon, UK

**Keywords:** melanocortin receptors, accessory proteins, adrenal cortex, familial glucocorticoid deficiency, obesity

## Abstract

The five melanocortin receptors (MCRs) named MC1R–MC5R have diverse physiological roles encompassing pigmentation, steroidogenesis, energy homeostasis and feeding behavior as well as exocrine function. Since their identification almost 20 years ago much has been learnt about these receptors. As well as interacting with their endogenous ligands the melanocortin peptides, there is now a growing list of important peptides that can modulate the way these receptors signal, acting as agonists, antagonists, and inverse agonists. The discovery of melanocortin 2 receptor accessory proteins as a novel accessory factor to the MCRs provides further insight into the regulation of these important G protein-coupled receptor.

## THE MELANOCORTIN SYSTEM

The melanocortin receptors (MCRs) are a family of five G protein-coupled receptors (GPCRs; MC1R–MC5R) expressed in diverse tissues, which serve discrete physiological functions. Early studies delineated a preliminary model where the receptors were activated by melanocortin peptides, derived from post-translational processing and proteolytic cleavage of the precursor protein proopiomelanocortin (Eipper and Mains, 1980). Receptor activation increased intracellular cyclic adenosine monophosphate (cAMP) levels triggering several downstream signaling pathways. Subsequent research into melanocortin signaling has added several additional tiers of complexity to this basic schema. It has emerged that many of the MCRs bind to endogenous peptides beyond the melanocortin peptides, which can act as agonists, antagonists, partial agonists, and even inverse agonists at these receptors (Cone, 2006). Furthermore, although the cAMP pathway continues to serve as the predominant readout of MCR function in many studies, numerous other intracellular signaling cascades for example the mitogen-activated protein kinase pathway have also been implicated in melanocortin signaling (Vongs et al., 2004; Sutton et al., 2005; Patten et al., 2007; Rodrigues et al., 2009). An additional feature of MCR signaling which has come to light over recent years is the presence of accessory proteins which regulate receptor function. These observations have broadened our understanding of MCR signaling, unveiling a highly specialized system, which on a cellular level enables individual cells to generate a complex co-ordinated response to the unique complement of ligands in their microenvironment (**Table 1**).

**Table 1:** The members of the melanocortin receptor family, expression, action, and phenotype of individuals with mutations.

Receptor	Main sites of expression	Physiological functions	Disease phenotype of patients with loss-of-function mutations	OMIM
MC1R	Melanocytes, macrophage	Pigmentation, inflammation	Increased risk of skin cancers	155555
MC2R	Adrenal cortex	Adrenal steroidogenesis	Familial glucocorticoid deficiency	202200
MC3R	Central nervous system (CNS), gastrointestinal (GI) tract, Kidney	Energy homeostasis, inflammation	Obesity	155540
MC4R	CNS, spinal cord	Energy homeostasis, appetite regulation, erectile function	Obesity	155541
MC5R	Lymphocytes, exocrine cells	Exocrine function, regulation of sebaceous glands	Decreased production of sebaceous lipids in mice	600042

### MC1R

MC1R is expressed in the melanocytes of the skin and hair follicles. Activation of MC1R results in a switch from red/yellow pheomelanin pigment production to the production of brown/black eumelanin pigment; it also promotes cell proliferation, DNA repair, and cell survival. Variants in MC1R are associated with red hair color, fair skin, and increased skin cancer risk (Valverde et al., 1995). Many of these variant receptors have been studied *in vitro* and their function, as measured by cAMP production in response to [Nle4,D-Phe7]-α-MSH (NDP-MSH), has been shown to be impaired compared to wild-type. Until recently MC1R was thought to be exclusively activated by alpha-MSH endogenously produced by keratinocytes in response to ultraviolet radiation. Human β-defensin 3 has since emerged as a novel endogenous MC1R ligand whose binding appears to initiate a discrete complement of intracellular signaling pathways *in vitro* (Beaumont et al., 2012; Swope et al., 2012). The physiological importance of this interaction remains to be seen. MC1R is also expressed in macrophages and in adipocytes where its role is less clearly defined. At extremely high plasma adrenocorticotropin hormone (ACTH) concentrations, ACTH activation of MC1R leads to hyperpigmentation observed in patients with familial glucocorticoid deficiency (FGD) (Turan et al., 2012). The study of the genetic determinants of coat color in animal models led to the discovery of agouti (also known as agouti signaling peptide), a high-affinity antagonist of the MC1R (Lu et al., 1994). The identification of an endogenous, physiologically relevant GPCR antagonist marked the beginning of a paradigm shift in our understanding of GPCR signaling. Later work demonstrated the complexity of this molecule by exploring its ability to act as an inverse agonist (Vage et al., 1997), inhibiting constitutively active MC1R.

### MC2R

MC2R is predominantly expressed in the adrenal gland where it promotes the expression of steroidogenic enzymes in response to binding plasma ACTH. Mutations in MC2R result in FGD (Clark et al., 1993; Tsigos et al., 1993). This is a rare, life-threatening autosomal recessive disorder of adrenal resistance to ACTH wherein affected individuals have low serum levels of cortisol despite the presence of extremely high circulating levels of plasma ACTH. Patients typically present within a few months of life with symptoms of cortisol deficiency including recurrent infections, hypoglycemia, convulsions, failure to thrive, and shock. Milder forms of the disease have been observed to present later in life (Metherell et al., 2009; Hughes et al., 2010; Meimaridou et al., 2012). The classical presentation of FGD comprises isolated perturbation of the glucocorticoid axis; however, a small number of cases have been described where this is accompanied by a disorder of mineralocorticoid secretion (Lin et al., 2007; Chan et al., 2009a).

Mutations in MC2R account for 25% of cases of FGD. The remaining 75% of patients with FGD in whom mutations in MC2R have been excluded have formed the subject of genetic approaches to elucidate other causative loci. To date, mutations in three further genes have been associated with FGD. Mutations in the melanocortin 2 receptor accessory protein (MRAP), a single pass transmembrane protein implicated in MC2R function account for approximately 15–20% of cases of FGD (Metherell et al., 2005). Mutations in steroidogenic acute regulatory (STAR) protein are known to give rise to lipoid congenital adrenal hyperplasia, a severe form of adrenal insufficiency characterized by both glucocorticoid and mineralocorticoid deficiency together with gonadal deficiency; however, STAR mutations have also been identified in a number of patients with FGD suggesting that mutations in this gene can give rise to a spectrum of clinical phenotypes encompassing FGD (Metherell et al., 2009). Recently, mutations in nicotinamide nucleotide transhydrogenase (NNT) a mitochondrial membrane constituent which is involved in detoxification of reactive oxygen species were also associated with FGD (Meimaridou et al., 2012). Notably, mutations in minichromosome maintenance-deficient 4 (MCM4) which forms part of a protein complex which is essential for DNA replication and genome stability are associated with a variant of FGD found in the Irish Traveller population where adrenal failure is accompanied by short stature, chromosome instability, and natural killer cell dysfunction (O’Riordan et al., 2008; Gineau et al., 2012; Hughes et al., 2012).

### MC3R

MC3R is primarily expressed in the central nervous system where it is found in the hypothalamus and the limbic regions (Roselli-Rehfuss et al., 1993). Targeted deletion of MC3R in murine models results in animals with increased fat mass, reduced lean mass, and reduced physical activity in the absence of hyperphagia (Butler et al., 2000; Chen et al., 2000). Additionally, these animals exhibit accelerated weight gain when placed on a high fat diet which is independent of hyperphagia implying that MC3R may be involved in nutrient partitioning. An emerging aspect of the MC3R deficient phenotype is that when these animals are subjected to food restriction regimes they exhibit impaired synchronized oscillation of the transcription factors which regulate liver clock activity and metabolism, utilize carbohydrates inappropriately, and fail to demonstrate the food anticipatory behaviors which are seen in wild-type mice (Sutton et al., 2008, 2010). These observations have led to the suggestion that MC3R is involved in coordinating appropriate homeostatic metabolic and behavioral responses to nutrient cues. Interestingly, despite focused investigation, rare variants at the MC3R locus have not been definitively associated with monogenic obesity in humans (Seng Lee et al., 2007; Mencarelli et al., 2011).

### MC4R

MC4R is widely expressed in the central nervous system where it is abundant in several regions including the paraventricular nucleus (PVN) of the hypothalamus (Mountjoy et al., 1994). Targeted deletion of MC4R in a mouse model results in animals which are severely obese, with hyperphagia and reduced energy expenditure (Huszar et al., 1997). In an informative study, Cre-lox technology was used to selectively re-express MC4R at endogenous levels in the PVN and the amygdale of MC4R knockout animals (Balthasar et al., 2005). This was able to partially rescue their obese phenotype by normalizing their food intake suggesting that MC4Rs in these regions are responsible for regulating eating behavior while MC4Rs in anatomically distinct neuronal populations are relevant to its control of energy expenditure.

The PVN is a key integration center for diverse signals which impact upon energy balance and is densely innervated by the arcuate nucleus of the hypothalamus. The release of proopiomelanocortin (POMC)-derived peptides from neurons in the arcuate nucleus, which are stimulated by leptin results in the activation of MC4R signaling in the PVN (Cheung et al., 1997; Schwartz et al., 1997). Uniquely for a biological system, a physiologically relevant endogenous antagonist and inverse agonist of MC4R (as well as MC3R) signaling is also produced in the hypothalamus, agouti-related peptide (AGRP; Nijenhuis et al., 2001; Breit et al., 2006). ARGP is produced by leptin responsive neurons in the arcuate nucleus that are inhibited by leptin. The presence of endogenous agonists and antagonists for this receptor enables MC4R signaling in the PVN to be delicately balanced.

Mutations in the MC4R are the most prevalent form of monogenic obesity identified to date accounting for up to 6% of patients with severe obesity (Vaisse et al., 1998; Yeo et al., 1998). The signaling capacities of specific mutant receptors studied *in vitro* correlates with the severity of the phenotype of corresponding MC4R deficient individuals (Farooqi et al., 2003). This demonstrates that the system is sensitive to degrees of loss in function at the level of the receptor. In addition to rare variants which cause highly penetrant forms of monogenic obesity, common variants in MC4R have been associated with body mass index (BMI) in genome wide association scans suggesting that variation at this locus also contributes to obesity in the general population (Willer et al., 2009).

### MC5R

The MC5R is widely expressed in peripheral tissues. Mice lacking MC5R are unable to produce the full complement of sebaceous lipids which constitute the water repellent component of their coats (Chen et al., 1997). As a result they demonstrate impaired water repulsion and thermoregulation. Studies in these mice have also suggested that MC5Rs play a role in the generation of pheromones which in turn influences aspects of behavior (Morgan et al., 2004; Morgan and Cone, 2006). High levels of MC5R are found in multiple exocrine tissues where they are thought to regulate the synthesis and secretion of a diverse range of exocrine products.

## THE MELANOCORTIN RECEPTOR ACCESSORY PROTEINS

### THE DISCOVERY OF MRAP

The functional properties of the MCRs have been extensively examined in *in vitro* expression systems. Comparable studies of MC2R function were initially impeded by difficulties in expressing functional receptor in conventionally used cell lines. It became apparent that unlike other MCRs functional MC2R could only be expressed in cell lines of adrenal origin, suggesting that its expression was contingent upon the presence of a tissue-specific protein. This factor was identified in a study of genetic loci associated with FGD. Mutations in MC2R were known to lead to this inherited syndrome of ACTH resistance, but were only able to account for approximately 25% of cases. Whole genome single nucleotide polymorphism (SNP) analysis in two affected consanguineous families in whom mutations in MC2R had been excluded identified a region of interest encompassing a novel protein, which was highly expressed in the adrenal gland. *In vitro* studies confirmed that expression of this protein alongside MC2R enabled functional MC2R receptor expression in heterologous cell types and it was subsequently named MRAP (Metherell et al., 2005). Fifteen to twenty percent of cases of FGD are now known to be caused by mutations in MRAP.

### MRAP STRUCTURE

**FIGURE 1:**
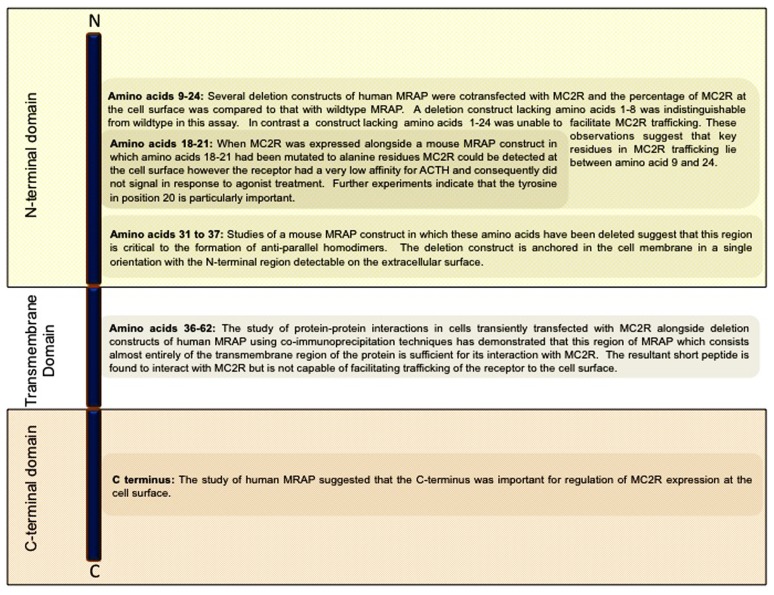
**Schematic representation of the domain structure of MRAP highlighting regions of functional significance in MRAP dimerization and in its interaction with MC2R (Sebag and Hinkle, 2009b; Webb et al., 2009)**.

Human MRAP is a single pass transmembrane protein which exists as two isoforms which differ in their C-termini. Both isoforms have been shown by reverse transcription polymerase chain reaction (RT-PCR) in a cDNA panel derived from human tissues to be expressed at high levels in the adrenal gland (Metherell et al., 2005). MRAP expression has also been demonstrated in a variety of other human tissues including testis, breast, thyroid, lymph nodes, ovary, and skin (Metherell et al., 2005). Immunofluorescence microscopy staining of cultured cells which endogenously express MRAP, using antibodies raised against N-terminal and C-terminal MRAP peptides, have shown that both regions of MRAP are present at the cell surface (Sebag and Hinkle, 2007). *In vitro* studies where differentially epitope-tagged forms of the protein were co-expressed in cells and their interaction and orientation were studied show that MRAP monomers form unique anti-parallel homodimeric structures (Sebag and Hinkle, 2007; Cooray et al., 2008). These dimers can be visualized in the endoplasmic reticulum and at the plasma membrane in live cells using biomolecular fluorescence complementation techniques (Sebag and Hinkle, 2009b, 2010). The deletion of amino acids 31–37 in mouse MRAP which directly precede the transmembrane portion of the protein abolishes its ability to homodimerize suggesting that this region of the protein constitutes a critical component of the interface between the two monomers (Sebag and Hinkle, 2009b). This mutant is unable to assist trafficking of the MC2R to the cell surface, suggesting that this unique conformation is essential to certain aspects of MRAP function (**Figure 1**).

## MRAP AND MC2R

Cell lines derived from adrenocortical tissue express both MC2R and MRAP and generate cAMP in response to treatment with ACTH. This response is abrogated by MRAP knockdown in these cells (Cooray et al., 2008). MRAP expression is necessary and sufficient for the functional expression of MC2R constructs in heterologous cell lines such as chinese hamster ovary (CHO) cells and SK-N-SH cells which are derived from ovarian and neuroblastoma tissue, respectively and do not endogenously express measurable levels of MRAP (Metherell et al., 2005; Sebag and Hinkle, 2007).

### MRAP FACILITATES TRAFFICKING OF THE MC2R TO THE CELL SURFACE

When epitope-tagged MC2R is expressed in CHO cells it is visualized in the endoplasmic reticulum, however, when it is expressed alongside MRAP it is predominantly localized to the plasma membrane (Metherell et al., 2005; Sebag and Hinkle, 2007; Webb et al., 2009). Consistent with this finding, in cells of adrenal origin with endogenously expression of MRAP, MC2R can be demonstrated at the cell surface. Western blotting of cell lysates yields a different complement of MC2R species in the presence and absence of MRAP, suggesting that MRAP may enhance receptor trafficking to the cell surface by promoting post-translational modification (Sebag and Hinkle, 2007). Deletion mutants of the N-terminal region of human MRAP which lack amino acids 1–24 or 1–35 are unable to promote MC2R trafficking to the cell surface suggesting that the N-terminal region plays a prominent role in receptor trafficking (Webb et al., 2009).

### MRAP FACILITATES MC2R SIGNALING

*In vitro* experiments examining MC2R signaling in the presence of various MRAP mutants suggest that the ability of MRAP to promote MC2R trafficking to the cell surface does not wholly account for its role in facilitating MC2R signaling. Amino acids within the N-terminal region of MRAP appear to influence MC2R signaling independently of enhancing receptor trafficking. Specifically, MC2R receptor is able to traffic to the cell surface but is unable to bind to ACTH in the presence of a deletion mutant of mouse MRAP in which amino acids 18–21 have been mutated to alanine residues (Sebag and Hinkle, 2009b).

### MRAP AND MC2R COMPLEXES

**FIGURE 2:**
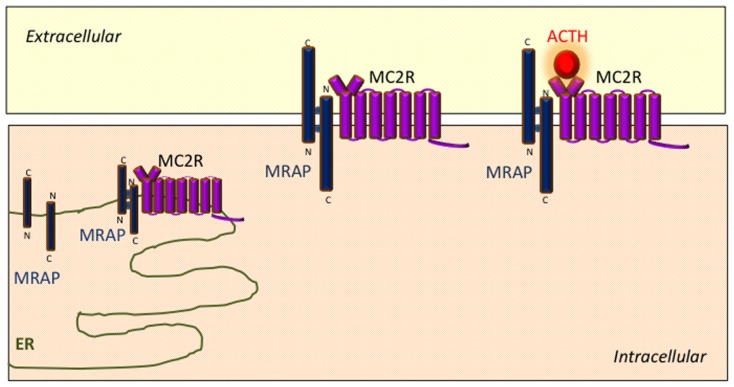
**Schematic representation of the role of MRAP in MC2R signaling**. MRAP monomers form unique anti-parallel homodimeric complexes in the endoplasmic reticulum which interact with MC2R and assist its trafficking to the cell surface. The MRAP/MC2R structure is capable of binding ACTH at the cell surface and initiating intracellular signaling.

Protein complexes containing both MRAP and MC2R can be reciprocally isolated using co-immunoprecipitation techniques in transiently transfected cells (Metherell et al., 2005; Webb et al., 2009). Analogous experiments using MRAP deletion mutations indicate that the transmembrane region of MRAP interacts with MC2R (Webb et al., 2009). A model has emerged wherein MRAP–MC2R complexes interact with one another to form higher order complexes which facilitate MC2R function. BRET techniques have been used to explore some of the interactions within these complexes in more detail in live cultured cells with specific reference to the effect of the MC2R agonist ACTH. These experiments have shown that ACTH enhances the interaction between MC2R homodimers and MRAP–MC2R heterodimers (Cooray et al., 2011). Schematic representation of the role of MRAP in MC2R signaling is shown in **Figure 2**.

## MRAP INTERACTIONS WITH OTHER MEMBERS OF THE MELANOCORTIN RECEPTOR FAMILY

MRAP can be demonstrated to form protein complexes with all five members of the MCR family using co-immunoprecipitation techniques in cultured cells (Chan et al., 2009b). Aside from MC2R, the remaining members of the MCR family are capable of efficiently trafficking to the cell surface when expressed in CHO cells. In a series of experiments using human constructs, co-expression of MRAP alongside either MC1R or MC3R in CHO cells was not found to alter cell surface expression of the receptors or influence their ability to generate cAMP in response to agonist treatment. In contrast, the cell surface expression and signaling capacities of both MC4R and MC5R were found to be significantly reduced in the presence of MRAP in these cells (**Table 2**). Signaling in these experiments was assessed by measuring cAMP generation using a competitive protein binding assay in response to treatment with a single concentration of agonist (Chan et al., 2009b). When the signaling properties of MC4R in the presence of MRAP were studied by measuring cAMP generation with a luciferase assay across a range of agonist concentrations using mouse constructs, co-expression of MRAP was not found to influence MC4R signaling (Sebag and Hinkle, 2010). Further examination of this interaction is needed to definitively understand whether MRAP interacts with MC4R *in vivo* and to determine the impact of this interaction on MC4R signaling. The *in vitro* interaction of MRAP and MC5R has been explored more fully using bimolecular fluorescence complementation techniques (Sebag and Hinkle, 2009a). When MC5R constructs are expressed in CHO cells MC5R monomers interact to from homodimers or oligomers. When MRAP is expressed alongside MC5R fewer multimeric complexes are formed and fewer receptors are expressed at the cell surface. This data suggests that MRAP is capable of interacting with members of the MCR family beyond MC2R and may differentially regulate aspects of their function. However, patients with MRAP mutations present with isolated glucocorticoid deficiency apparently without any other additional symptoms (Chung et al., 2010). One patient with FGD caused by a mutation in MRAP was described as being concomitantly obese, although the significance of this is unclear as the rest of the family had similarly high BMIs (Rumie et al., 2007).

**Table 2:** *In vitro* effects of MRAP on melanocortin receptors, based on data derived from Metherell et al. (2005) Sebag and Hinkle (2007, 2009a, 2010), Chan et al. (2009b) and Cooray et al. (2011).

Receptor	Effect of MRAP on trafficking	Effect of MRAP on signaling (cAMP generation)
MC1R	No change in cell surface expression	No effect in response to NDP-MSH
MC2R	Required for surface expression and receptor maturation	Required of receptor signaling in response to ACTH
MC3R	No change in cell surface expression	No effect in response to NDP-MSH
MC4R	Reduction in the surface expression	Reduction in response to NDP-MSH (No change was seen in another study)
MC5R	Reduction in the surface expression	Reduction in response to NDP-MSH

## MRAP IN ADIPOCYTES

Prior to its advent as an accessory factor for MC2R in the adrenal gland, MRAP was identified as a novel protein expressed in mouse adipocytes (Xu et al., 2002). Both isoforms of MRAP have subsequently been shown by RT-PCR to be expressed in human adipocyte tissue (Metherell et al., 2005). The function of MRAP in these cells is not known. Given its established role in facilitating MC2R function in the adrenal gland it is likely that its function is related to the melanocortin system. The distribution of epitope-tagged MRAP within cultured adipocytes is responsive to insulin treatment suggesting that its role is contingent upon metabolic cues. Notably, gross metabolic dysfunctions have not been reported in patients with mutations in MRAP; however, this may reflect functional redundancy with the related protein MRAP2 in this tissue whose expression in adipocytes has not yet been investigated. The components of the melanocortin system which are expressed in adipocyte tissue show species-specific differences. 3T3L1 cells which are cells of murine origin that are commonly used in the study of adipocyte function express both MC2R and MC5R. Human adipose tissue samples have been shown to express MC1R and low levels of MC4R and MC5R (Hoch et al., 2007). Treatment of 3T3L1 cells with melanocortin ligands promotes lipolysis and cytokine production *in vitro*, however, this observation has not been replicated in human mesenchymal cell derived adipocytes (Hoch et al., 2008; Jun et al., 2010; Moller et al., 2011).

## IDENTIFICATION OF MRAP2

Following the discovery of MRAP a related gene of unknown function with a comparable structure and a high degree of N-terminal and C-terminal sequence similarity was identified on chromosome 6 and was named MRAP2 (Metherell et al., 2005; Chan et al., 2009b). MRAP2 is a single pass transmembrane protein, in humans it has been shown by RT-PCR to be expressed in the brain and the adrenal gland (Chan et al., 2009b). Western blotting of mouse tissues resolves a single band of MRAP2 reactive species whose molecular weight corresponds to a dimer suggesting that endogenous MRAP2 exists in dimeric complexes (Chan et al., 2009b). MRAP2 undergoes N-linked glycosylation at its N-terminus and is thought to form anti-parallel homodimeric complexes analogous to those formed by MRAP (Chan et al., 2009b; Sebag and Hinkle, 2009a, 2010). MRAP2 constructs can be visualized in the endoplasmic reticulum and the plasma membrane of transiently transfected CHO cells (Chan et al., 2009b).

## MRAP2 AND MC2R SIGNALING

The physiological role of MRAP2 is not yet known. In view of the structural similarities between MRAP and MRAP2 and its concurrent expression in the adrenal gland, the interaction between MRAP2 and MC2R has been explored in detail. As discussed previously, when MC2R constructs are expressed in CHO cells in isolation the receptors are unable to reach the cell surface. Concurrent expression of MRAP facilitates MC2R trafficking to the cell surface. At the cell surface MC2Rs are activated by ACTH binding and generate a cAMP response, the magnitude of which is dependent on the ambient ligand concentration. Expression of MRAP2 alongside MC2R promotes MC2R trafficking to the cell surface to a similar extent as MRAP; however, the resultant complex has an extremely low affinity for ACTH binding. As a result the response curve of cAMP generated plotted against ligand concentration is significantly shifted to the right when MC2R is co-expressed with MRAP2 compared to when MC2R is expressed with MRAP (Sebag and Hinkle, 2009a, 2010; Gorrigan et al., 2011). It was suggested that this difference was due to lack of the leucine, aspartic acid, tyrosine, isoleucine (LDYI) motif in MRAP2, which was present in MRAP, and insertion of the LDYI residues enabled the MRAP2/MC2R complex to respond to lower concentrations of ACTH (Sebag and Hinkle, 2009b). MRAP and MRAP2 are able to form heterodimeric complexes *in vitro*, however, whether such a complex is physiologically relevant is not known (Chan et al., 2009b; Sebag and Hinkle, 2010). In one study MC2R was expressed alongside both MRAP and MRAP2, the resulting dose response curve appeared sensitive to the ratio of MRAP to MRAP2, with increasing proportions of MRAP2 shifting the curve further to the right (Sebag and Hinkle, 2010). Importantly, this effect has not been observed in other studies (Chan et al., 2009b; Agulleiro et al., 2010). Interestingly, Y1 cells which are of murine adrenocortical origin and endogenously expresses MRAP, MRAP2 and MC2R generate cAMP in response to ACTH treatment and this effect can be abrogated by transient expression of MRAP2 within these cells (Sebag and Hinkle, 2010). Taken together, further work is required to determine if the expression levels of MRAP and MRAP2 in the adrenal gland could act as a physiological mechanism dictating ACTH responsiveness.

Mutations in MC2R and MRAP lead to the inherited condition FGD. In contrast, mutations in the MRAP2 gene have not been linked with this condition suggesting that it may not be involved in MC2R signaling *in vivo* and that any degree of functional redundancy between MRAP and MRAP2 is unable to completely compensate for MRAP dysfunction. In-keeping with this observation *in situ* hybridization studies and quantitative RT-PCR using rat tissue have demonstrated that MRAP is expressed at much higher levels than MRAP2 in adult adrenal glands (Gorrigan et al., 2011).

## MRAP2 BEYOND MC2R SIGNALING

Consistent with the findings discussed above, exploring the interaction of MRAP with other members of the MCR family, MRAP2 has also been shown to interact with all five members of the MCR family by co-immunoprecipitation assays in transiently transfected cultured cells (Chan et al., 2009b). In one series of experiments when cell surface expression of individual MCRs was quantified in the presence or absence of MRAP2, expression levels of MC1R and MC3R were not affected by MRAP2 expression, however, expression levels of MC4R and MC5R were significantly reduced when they were co-expressed with MRAP2. Furthermore, when MCR signaling was examined in the presence of MRAP2, its expression was detrimental to MC3R, MC4R, and MC5R signaling but had no effect on MC1R signaling. In these experiments signaling was studied by quantifying cAMP generation in response to treatment with a single concentration of ligand. The effect of MRAP2 on MC4R signaling has been explored using a luciferase reporter assay to measure cAMP production across a range of ligand concentrations. In this study the authors concluded that although MC4R surface expression was reduced in the presence of MRAP2 that MC4R signaling did not appear to be affected (Sebag and Hinkle, 2010). The experiments discussed here have been conducted in CHO cells which do not endogenously express any of the components of the melanocortin system, further research will address whether MRAP2 interacts with MCRs beyond this experimental system and will further delineate the nature of these interactions. As the main site of MRAP2 expression is in brain and specifically in the hypothalamus it is likely that the main physiological function of MRAP2 is in the central nervous system, perhaps involving MC4R and/or MC3R.

## THE FUTURE OF MRAPs

Numerous lines of genetic evidence suggest that subtle perturbations of signaling capacity at the level of MCRs can have pathological consequences. As our appreciation of the complex nature of the melanocortin system grows, a large family of molecules is emerging which impact upon signaling and confer the ability to integrate extracellular and intracellular cues into a co-ordinated biological signal. The discovery of two proteins which may interact with multiple members of the MCR family in distinct ways represents a significant advance in our understanding of melanocortin signaling and paves the way for further lines of research to explore the physiological roles of these proteins.

## Conflict of Interest Statement

The authors declare that the research was conducted in the absence of any commercial or financial relationships that could be construed as a potential conflict of interest.
